# The role of non-coding RNA in the diagnosis and treatment of *Helicobacter pylori*-related gastric cancer, with a focus on inflammation and immune response

**DOI:** 10.3389/fmed.2022.1009021

**Published:** 2022-10-13

**Authors:** Ao-ran Liu, Zi-wei Yan, Li-yue Jiang, Zhi Lv, Yan-ke Li, Ben-gang Wang

**Affiliations:** ^1^Tumor Etiology and Screening Department of Cancer Institute and General Surgery, The First Affiliated Hospital of China Medical University, Key Laboratory of Cancer Etiology and Prevention, China Medical University, Liaoning Provincial Education Department, Shenyang, China; ^2^Tangdu Hospital of the Fourth Military Medical University, Xi’an, China; ^3^Department of General Surgery, The First Hospital of China Medical University, Shenyang, China; ^4^Department of Hepatobiliary Surgery, Institute of General Surgery, The First Hospital of China Medical University, Shenyang, China

**Keywords:** *Helicobacter pylori*, non-coding RNA, *H. pylori*-related GC, inflammation, immune response, microRNA

## Abstract

*Helicobacter pylori* (*H. pylori*) is one of the globally recognized causative factors of gastric cancer (GC). Currently, no definite therapy and drugs for *H. pylori*-related GC have been widely acknowledged although *H. pylori* infection could be eradicated in early stage. Inflammation and immune response are spontaneous essential stages during *H. pylori* infection. *H pylori* may mediate immune escape by affecting inflammation and immune response, leading to gastric carcinogenesis. As an important component of transcriptome, non-coding RNAs (ncRNAs) have been proven to play crucial roles in the genesis and development of *H. pylori*-induced GC. This review briefly described the effects of ncRNAs on *H. pylori*-related GC from the perspective of inflammation and immune response, as well as their association with inflammatory reaction and immune microenvironment. We aim to explore the potential of ncRNAs as markers for the early diagnosis, prognosis, and treatment of *H. pylori*-related GC. The ncRNAs involved in *H. pylori*-related GC may all hold promise as novel therapeutic targets for immunotherapy.

## Introduction

Gastric cancer (GC) is one of the leading malignancies in terms of morbidity and mortality worldwide, which has attracted extensive attention among global health problems (1). Its high incidence and mortality are mainly attributed to some unclear pathogenesis. The chronic infection of *Helicobacter pylori* (*H. pylori*) has been well-accepted as one of the multiple pathogenic factors of GC. It has been listed as a primary carcinogen by the International Agency for Cancer (2, 3). Chronic *H. pylori* infection can result in diseases such as gastritis, gastric ulcer and GC over time, and gastric mucosa-associated lymphoid tissue (MALT) lymphoma may also develop in some patients (4–7). However, the specific mechanism of *H. pylori* infection on gastric carcinogenesis remains unidentified in spite of the known causality between them. Exploration for the mechanism would be conducive to GC prevention and treatment.

As a key element of pathogenic factors for *H. pylori*-related GC, the virulence factors of *H. pylori* associated with immune response mainly consist of cytotoxin-associated gene A (CagA), vacuolating cytotoxin A (VacA), and *H. pylori* neutrophil-activating protein (HP-NAP), etc. (8). Among them, CagA was reported to exist in more than 60% of *H. pylori* strains considered to be a strong activator of NF-κB and a major mediator of carcinogenesis (9). CagA can not only bind to NF-κB *via* beta-catenin but also independently affect NF-κB and produce inflammatory factors such as IL-8 to mediate immune response (9, 10). VacA inhibits capital histocompatibility complex class II (MHC II)-dependent pathways and releases pro-inflammatory factors including IL-1β, IL-6, IL-10, and tumor necrosis factor-alpha (TNFα) by forming vacuoles within macrophages, thus inducing immune escape and protecting *H. pylori* (11). Unlike VacA, HP-NAP up-regulates MHC II, promotes Th1 immune response, induces the expression of IL-12 and IL-23 in neutrophils and monocytes, and triggers ROS release to destroy epithelial cells (12, 13). The recognition of lipopolysaccharide (LPS) from *H. pylori* by toll-like receptors (TLRs, mainly TLR4 and TLR2) in human body could activate the NF-κB pathway and promote the chemotaxis of immune cells such as neutrophils and dendritic cells to release large amounts of inflammatory factors attempting to phagocytose *H. pylori*. However, the specific virulence factors of *H. pylori* could protect themselves from phagocytosis initiated by innate immune response. Consequently, the large quantity of inflammatory factors have to accumulate at *H. pylori* colonization sites and cause a long-term inflammation in epithelium, leading to chronic gastritis and even GC (14).

The unique virulence factors enable *H. pylori* to escape from the eradication by immune system and colonize in gastric mucosa making carcinogenic effects. After *H. pylori* infection, innate immunity is first activated to release pro-inflammatory factors and immune cells intending to phagocytose and eliminate *H. pylori*, then local immune microenvironment is altered. Once the changes fail to resist the pathogenic effects of virulence factors, *H. pylori* could colonize in gastric mucosa, break through the barrier and survive in the deep site of stomach chronically, resulting in gastritis, gastric ulcer and even GC over time. Therefore, inflammation and immune response are essential parts during the initiation of *H. pylori*-related GC.

Mounting studies about *H. pylori*-related GC have focused on non-coding RNAs (ncRNAs). NcRNAs consist of microRNAs (miRNAs), long ncRNAs (lncRNAs), circular RNAs (circRNAs), small nucleolar RNAs (snoRNAs), and piRNAs (15). In the early stage after discovery, ncRNAs were regarded as useless “garbage” in body due to the lack of capability to encode proteins. With the depth of research, however, the important regulatory function of ncRNAs in many basic cellular processes has been gradually revealed including development, differentiation, proliferation, transcription, post-transcriptional modification, immune regulation, cell apoptosis, and metabolism (16). Despite the inability of ncRNAs to directly encode proteins, more than 60% of their downstream target genes have the competence (17). It has been suggested that rare ncRNAs can independently function in diseases. They usually interact with each other to construct powerful networks and affect many proteins determining cell fates as well as specific cellular biological process by regulating functional stability. The complex interactions make the dysregulation of ncRNAs quite common in cancer (18–20). The immune inflammatory response occurring in *H. pylori*-related GC can alter the expression patterns of ncRNAs, thereby affecting the expression of downstream proteins or target genes and GC biological behaviors.

In the present review, we integrated the articles studying ncRNAs with immune inflammation in *H. pylori*-related GC, summarize the research frontiers of ncRNAs in this field and explore the potential of ncRNAs as diagnostic and prognostic markers for *H. pylori*-related GC, aiming to provide theoretical basis for further investigation of ncRNAs on the immune direction in *H. pylori*-related GC. The immunization of ncRNAs in *H. pylori*-related GC might give a new sight for GC diagnosis and treatment.

## MiRNAs and *Helicobacter pylori*-related gastric cancer

MiRNAs are short ncRNAs with approximately 22 nucleotides in length. They can inhibit the expression or function of downstream target genes by inducing degradation or translational inhibition through binding to the 3′ untranslated region (3′-UTR) of target genes (especially mRNAs) with their 5′ ends (21). MiRNAs are the most extensively studied ncRNAs so far. The association of miRNAs with *H. pylori*-related GC has been gradually emphasized on immune inflammation. TLRs could be activated by LPS from *H. pylori*, and aberrant TLRs may promote the expression of some miRNAs. MiRNAs can also affect the NF-κB pathway by regulating TLRs expression and the TLR signaling pathway in turn, inducing the release of pro-inflammatory factors, transcription factors and cascades (22, 23). The structure of LPS could also be modulated by small RNAs to influence the immune recognition in host thereby enhancing *H. pylori* resistance (24). Therefore, miRNAs are shown to have the potential to serve as detective markers for early diseases (25–28).

### MiR-155

MiR-155 has emerged as a key factor in innate immunity and inflammatory reaction. *H. pylori* could induce miR-155 in gastric epithelial cells and gastric mucosa, which has been widely reported to damage *H. pylori* by triggering immune response (23, 29–33). The induction of miR-155 in T cells mediated by *H. pylori* might be based on a cAMP-Foxp3-dependent manner (34). MiR-155 promotes the release of inflammatory factors including TNF-α, IL-6, and IL-23 in exosomes from macrophages infected with *H. pylori* and simultaneously increases the expression of CD40, CD63, CD81, and MCH-I, suggesting that miR-155 may regulate *H. pylori*-induced inflammation in host cells *via* exosomes (35). MiR-155 can also promote *H. pylori* elimination by inducing autophagy to enhance the sterilization ability of host to intracellular *H. pylori* (36). The association of miR-155 with immunity in *H. pylori* infection appears to be proved in mice. The generation of miR-155^–/–^ mice might be owing to an inherent defect in T cells that impairs specific Th1 and Th17 cells making them disabled to proliferate, produce IFN-γ and IL-17, thus control *H. pylori* infection effectively (37). In addition, miR-155 is up-regulated in macrophages during *H. pylori* infection dependent on TLR and type IV secretion system (T4SS), inhibiting cell apoptosis caused by DNA damage (38).

However, some researchers believed that miR-155 negatively regulated inflammatory reaction and reduced immune response of body to protect *H. pylori*. Xiao et al. (39) found that the induction of miR-155 by *H. pylori* was dependent on the activation of NF-κB and AP-1 pathways. After miR-155 overexpression, a down-regulation was demonstrated in IkappaB kinase epsilon (IKK-ε), Fas-associated death domain (FADD) as well as Sma- and Mad-related protein 2 (SMAD2), which negatively regulated the release of IL-8 and growth-related oncogene-α (GRO-α). Except for miR-155, myeloid differentiation protein 88 (MyD88) was also observed to be involved in the negative regulation of *H. pylori*-induced inflammatory reaction as a target gene of miR-155 (40).

The diverse roles of miR-155 in *H. pylori*-induced immune inflammatory response mentioned above indicated it as a potential marker for *H. pylori*-related GC.

### MiR-223

MiR-223 was also suggested to be involved in pathways associated with innate immunity and inflammatory reaction in patients with *H. pylori* infection (41–43). CagA of *H. pylori* may induce miR-223-3P expression through the NF-κB pathway. And miR-223-3p can directly target AT-rich interactive-domain 1A (ARID1A), a tumor suppressor protein with ATP enzymatic activity, to promote GC proliferation and migration. Therefore, *H. pylori* might participate in CagA-mediated biological effects in GC cells *via* the NF-κB/miR-223-3p/ARID1A axis (44). MiR-223-3p and IL-10 secreted by macrophages exert inhibitory effects on pro-IL-1β and inflammasome NLRP3 both secreted by monocytes during *H. pylori* infection (32, 45). Furthermore, the mucosal expression level of miR-223 was significantly decreased following with the disappearance of neutrophils from gastric mucosa in patients after *H. pylori* eradication (46). A recent study, however, showed that miR-233 expression decreased in *H. pylori*-associated autoimmune atrophic gastritis and multifocal atrophic gastritis. Hence, the anti-inflammatory function of miR-233 might vary with different microenvironments (47). These findings made miR-223 also a potential marker for the diagnosis or treatment of *H. pylori*-related GC.

### MiR-375

MiR-375 appears to be closely associated with immune inflammatory response in *H. pylori*-related GC. It was shown to be down-regulated after *H. pylori* infection accompanied by the activation of JAK2-STAT3 (48–51). Janus kinase 2 (JAK2) was identified as a target gene of miR-375, and miR-375 could negatively regulate the expression of programmed cell death-ligand 1 (PD-L1) in GC through the JAK2/STAT3 signaling pathway (50). *H. pylori* may affect the JAK2-STAT3 pathway releasing cytokines including IL-6, IL-10, and VEGF by down-regulating miR-375, inhibit the maturity of dendritic cells, reduce CD4^+^ and CD8^+^ T cells, then inhibit immune response mediating the immune escape of *H. pylori* to promote gastric carcinogenesis (52). Moreover, the regulation of JAK2-STAT3 signaling by miR-375 could release IL-1β, IL-6, IL-8, and TNF-α by activating downstream target genes BCL-2 and TWIST1, which promotes tumor transformation and gets involved in *H. pylori*-induced cell proliferation and migration (53). Rossi et al. found that the levels of IL-6, IL-12A, and IL-2 were significantly elevated in *H. pylori*-positive patients followed by the down-regulation of miR-103, miR-181c, miR-370, and miR-375 (54). All above-mentioned results suggested that miR-375 participated in *H. pylori*-related GC by regulating immune response.

### MiR-146

MiR-146 might be involved in the development of *H. pylori*-related gastric diseases *via* the NF-KB pathway. *H. pylori* infection could trigger inflammatory reaction of body and induce the production of IL-17A, GRO-α, IL-8, and miR-146α in GC cells successively. For its mechanism, IL-17A might mediate miR-146α to regulate inflammatory reaction in an NF-κB-dependent manner during *H. pylori* infection (55). Other than *H. pylori*-related GC, miRNAs especially miR-146a and miR155 also received much attention in *H. pylori*-related pediatric gastritis, which were likely to be associated with its prognosis (56, 57).

### Other miRNAs

Most miRNAs involved in the regulation of *H. pylori*-related GC had association with the NF-κB pathway. The decrease of miR-204 level induced by *H. pylori* infection could up-regulate the downstream target gene BIRC2, enhance the activity of BIRC2/TNF-α/NF-κB signaling pathway, thus promote angiogenesis and metastasis of GC cells, leading to poor prognosis of *H. pylori*-related GC patients (58). *H. pylori* infection was reported to induce miR-18a-3P and miR-4286 expression in GC through TLR4/NF-κB molecules related to immune recognition, inhibit the expression of downstream target gene BARAP1, thereby participate in innate immune response of body and inflammatory pathways associated with NF-κB (59). *H. pylori* activates NF-κB and reduces miR-218 expression to increase the level of downstream target gene called epidermal growth factor receptor-amplified and overexpressed protein (ECOP) promoting cell proliferation. Accordingly, Gao et al. believed that miR-218 could be considered as a therapeutic target for *H. pylori*-related GC. The inhibition of NF-κB by increasing miR-218 expression level artificially might become a kind of therapy to prevent the progression from precancerous lesion to cancer (60). In gastric epithelial cells infected with *H. pylori*, Tip-α inhibits miR-3178 targeting TRAF3 to increase TNF-α and IL-6 activating NF-κB to promote GC cell proliferation (61). Let-7b targeting TLR4 was shown to decrease in gastric epithelial cells infected with *H. pylori*. The knockdown of TLR4 concurrent with let-7b overexpression could reduce the expression of downstream genes associated with immune inflammatory response including NF-κB, MyD88, NF-κB1/p50, and RelA/p65 (62). Lin et al. found that serum miR-130b level was elevated in human and mice after *H. pylori* infection activating the NF-κB pathway positively correlated with myeloid-suppressor Schlafen4 (Slfn4). Further research revealed that gastric SLFN4^+^ cells infected with *H. pylori* might induce miR-130b and exhibit a T cell suppressive phenotype resulting in *H. pylori*-induced gastric metaplasia, tumor formation and growth (63). Based on these findings, *H. pylori* infection may activate the NF-κB pathway by regulating miRNAs to affect biological function facilitating the development of inflammation and cancer.

Additionally, miRNAs may also influence *H. pylori*-related GC by regulating PD-1/PD-L1 involved in immune response. PD-L1 might be a downstream target of miR-140. MiR-140 could increase IFN-γ and TNF-α levels, inhibit the PD-L1 and mTOR signaling pathways, and raise CD8^+^ T cells, thus inhibiting the proliferation of *H. pylori*-positive GC to exert anti-cancer roles (64). Xie et al. (65) reported that *H. pylori* could regulate B7-H1 binding to PD-1 by inhibiting miR-152 and miR-200b in GC cells, thereby suppress T cell proliferation and immune response. *H. pylori* might also alter the levels of miR-326 and miR-663 in CD4^+^ T cells (66).

Methylation has been suggested to be another mechanism by which miRNAs affect *H. pylori* through immune inflammatory response. The extract of *Celastrus orbiculatus* (COE) regulates *H. pylori*-induced inflammatory response by inhibiting miR-21 expression and the methylation level of target gene programmed cell death 4 (PDCD4) (67). Furthermore, the tumor suppressor factor miR-124 silences spermine oxidase (SMOX) in *H. pylori*-related GC *via* DNA methylation (68). JARID1B is a histone demethylase and its up-regulation is associated with immune cell infiltration in *H. pylori*-related GC. Therefore, the miR-29c/JARID1B/cyclinD1 axis could be a novel therapeutic pathway for GC (69).

**FIGURE 1:**
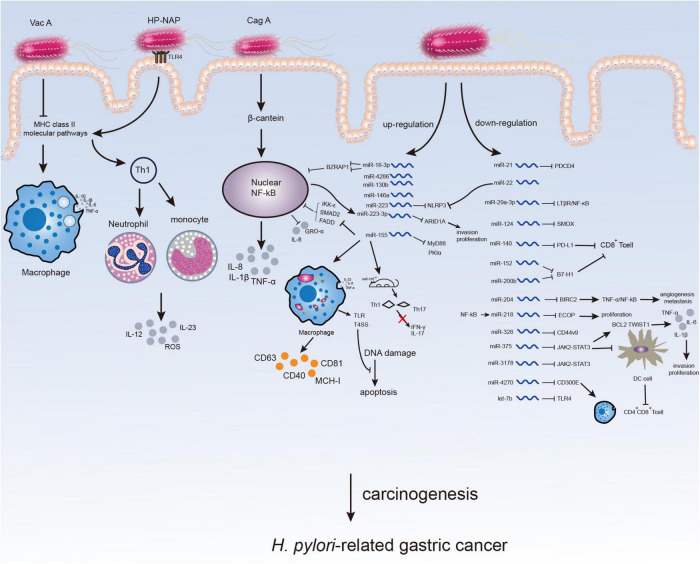
Microenvironment changes in *H. pylori*-infected gastric mucosa. The virulence factors of *H. pylori* can affect immune response or inflammation of host inducing *H. pylori*-related gastric carcinogenesis. The participation of *H. pylori* may not only directly through virulence factors but also by altering the expression of ncRNAs especially miRNAs, which might serve as potential therapeutic targets for *H. pylori*-related GC.

Some other miRNAs were also indicated to be associated with immunity or inflammation in *H. pylori*-related GC. *H. pylori* up-regulates the immune receptor CD300E by down-regulating miR-4270 to enhance the pro-inflammatory potential and impair the sterilization ability of macrophages (70). Chronic inflammation caused by *H. pylori* infection was shown to increase CD44V9 by down-regulating miR-328 in gastric mucosa (71). Except for miR-223-3p mentioned above, miR-22 can also directly target NLRP3, reduce inflammasomes levels and maintain the homeostasis of gastric microenvironment. *H. pylori* disrupts microenvironment homeostasis by inhibiting the regulation of miR-22 on downstream factor NLRP3 (72). In conclusion, miRNAs could regulate the expression of inflammatory factors and suppress immune response after *H. pylori* infection, contributing to the immune escape of *H. pylori* and ultimately GC (Table 1 and Figure 1).

**TABLE 1:** The regulation of miRNAs on inflammation and immune response in *H. pylori*-related gastric cancer.

MicroRNA(s)	Expression	Patients	Cell line	Target	Mechanism of action	References
miR-155	Up		RAW264.7		Promoted the expression of TNF-a, IL-6, IL-23, CD40, CD63, CD81, MCH-I, MyD88, and NF-κB	(35)
miR-155	Up				miR-155 -/- mice produce fewer IFN-γ and IL-17 than wt-T cells and do not differentiate into Th1 or Th17 cells, and do not cause immunopathology.	(37)
miR-155	Up	22	GES-1, AGS, MKN45 (HEK) 293	IKK-ε, SMAD2, and FADD	Down-regulate NF-κB and AP-1 pathway to negatively regulated IL-8 and GRO-α.	(39)
miR-155	Up		MKN74, AGS	The protein kinase A inhibitor alpha (PKIalpha)	miR-155 -cAMP-Foxp3 axis in T cells	(34)
miR-155	Up		GES-1		Induce the autophagy to decrease the survival of intracellular *H. pylori*	(36)
miR-155	Up		AGS and (HEK) 293 cells	MyD88	Reduce IL-8 production induced by *H. pylori* infection.	(40)
miR-223	Up	22	AZ-521		Increase the neutrophil and/or mononuclear cell infiltration	(46)
miR-223-3p	Up	50	SNU, AGS, MGC-803, and MKN1	ARID1A	NF-κB/miR-223-3p/ARID1A axis is involved in CagA-induced cell proliferation and migration	(44)
miR-223	Up		THP-1, AGS	NLRP3	Increase the copious amount of IL-10, IL-1β	(45)
miR-22	Down			NLRP3	Riggers’ uncontrolled proliferation of epithelial cells and the emergence of GC	(72)
mir-375	Down		BGC823, GES-1, and MFC	JAK2-STAT3	Promote the secretion of IL-6, IL-10, and VEGF, leading to immature differentiation of DCs and induction of gastric cancer.	(52)
miR-375	Down		BGC-823, AGS, SGC-7901, and MKN-45	JAK2	miR-375/JAK2-STST3 is involved in *H. pylori*-induced inflammation to induce IL-8 and TNF-α and promotes neoplastic transformation by affecting the expression of BCL-2 and TWIST1	(53)
miR-375	Down	31			Up-regulation of TNFA, IL6, IL12A, IL2, and TGF-β-RII.	(54)
miR140	Down	15	AGS, MGC803, SGC7901, BGC823, MKN45	PD-L1	Suppress GC by targeting immune checkpoint molecule PD-L1.	(64)
miR-200b	Down	76	AGS	B7-H1	*H. pylori* promoted B7-H1 expression which binds to PD-1 and inhibited miR-152 and miR-200b expression to promote gastric cancer	(65)
miR-152						
miR-204	Down	26	AGS, BGC-823, SGC-7901, and MGC-803	BIRC2	miRNA-204 leads to enhanced BIRC2 expression level and BIRC2/TNF-a/NF-kB signaling pathway activities, which promoted angiogenesis and metastasis of gastric cancer cells.	(58)
miR-18a-3p	Up		AGS, N87, and MKN45	BZRAP1	miR-18a-3p and miR-4286 activated the NF-κB transcription factor to increase cancer cell proliferation and motility and both inhibited expression of BZRAP1, but TAK-242 (TLR4 inhibitor) blocked this effect.	(59)
miR-4286						
miR-218	Down		AGS and MKN45	ECOP	miRNA-218 inhibits NF-κB activation by decreasing ECOP expression, increasing cell proliferation, and inhibiting cell apoptosis.	(60)
miR-146a	Up		SGC-7901		miR-146a inhibits the inflammatory responses induced by IL-17A during the infection of Hp	(55)
miR-3178	down		GES-1, SGC7901, and MGC803	TRAF3	Tip-α might activate NF-κB to promote inflammation such as TNF-α and IL-6, and carcinogenesis by inhibiting miR-3178 expression, which directly targets TRAF3	(61)
miR130b	Up	21–63			MiR130b induce T-cell suppressor phenotype and promoted Helicobacter-induced metaplasia	(43)
miR-21	Down			PDCD4	COE could inhibit microRNA-21 (miR-21) expression and target PDCD4 and induce inflammatory factors such as IL-6, IL-8, and TNF-α.	(67)
miR-124	Down		AGS	SMOX	miR-124 through the inhibition of SMOX-mediated DNA damage in the etiology of H. pylori-associated gastric cancer.	(68)
miR-4270	Down	10		CD300E	HP modulating the expression of the immune receptor CD300E through miR-4270	(70)
miR-328	Down	54	AGS.AGS cells were treated with TNF-α, interleukin-1b (IL-1b), or H2O2	CD44v9	High CD44v9 expression is significantly associated with low miR-328 expression can avoid cell death caused by various stress inducers and inhibit gastric cancer development.	(71)
miR-29a-3p	Down	82	GES-1, MGC-803, AGS, MKN-45, SGC-7901 and HGC-27	LTβR/NF-κB	HOXA-AS3/miR-29a-3p/LTβR/NF-κB regulatory axis contributes to the progression of GC	(83)
let-7	Down			TLR4	let-7b downregulate TLR4 and attenuated NF-κB, MyD88, NF-κB1/p50, RelA/p65, IL-8, COX-2, and CyclinD1	(62)

## LncRNAs and *Helicobacter pylori*-related gastric cancer

LncRNAs are long ncRNAs with more than 200 nucleotides in length. They play vital roles in cellular process despite not encoding proteins (73, 74). LncRNAs have been widely proven to be associated with GC genesis and development by affecting biological function (75).

It has been preliminarily explored for the regulatory relationship with mechanisms of lncRNAs in *H. pylori*-related GC. However, few studies have focused on immunity and inflammation, which is ought to be future research direction in this field. LncRNAs were demonstrated to be implicated in viral infection recently (76). In *H. pylori*-infected GC, a decreased expression of TNF-α, IL-1β, and IL-8 was detected after knockdown of plasmacytoma variant translocation 1 (lncPVT1) and the migration of GC cells was inhibited, suggesting that lncPVT1 might activate immune function to affect GC caused by *H. pylori* (77). Similar to this result, another study reported that the overexpression of lncRNA H19 induced inflammatory reaction *via* the NF-κB pathway, released pro-inflammatory cytokines including TNF-α, IL-1β, IL-6, and IL-8, then promoted GC cell proliferation, migration and invasion infected with *H. pylori* (78). Besides, circulating lncRNA H19 expression was significantly increased in *H. pylori*-positive peptic ulcer patients and further increased in GC patients statistically positively correlated with the levels of TNF-α, IFN-γ, and gastrin. Hence, lncRNA H19 could be applied to distinguishing GC from peptic ulcer with positive *H. pylori* (79). LncRNA SGK1 was found to be elevated in T cells of *H. pylori*-related GC, induce Th2 and Th17 cells, reduce Th1 cell differentiation through the SGK1/JunB signaling pathway and be associated with poor prognosis of *H. pylori*-infected GC (80). LncRNAs may also affect the progression of *H. pylori*-related MALT. It was shown that lncRNA GHRLOS exhibited significant change in gastric MALT patients, which had differential expression both in *H. pylori*-positive gastritis and GC tissue (81). That might be a sound evidence for lncRNAs as important factors in the development of gastric malignancies. The above findings indicated that lncRNAs might participate in gastric malignant tumors induced by *H. pylori* with the potential to be predictive biomarkers.

Other than independent effects, lncRNAs can also interact with miRNAs forming ceRNA networks to jointly regulate *H. pylori*. MiR-375 has been identified as an inhibitor of *H. pylori*-related GC, and the expression of lncRNA SOX2OT was down-regulated after miR-375 overexpression, suggesting the relationship of co-regulation between lncRNAs and miRNAs on *H. pylori*-related GC (82). LncRNA HOXA-AS3 is elevated in GC relevant with *H. pylori* infection. It negatively regulates miR-29a-3p and inhibits the downstream target gene LTβR regulating the NF-κB pathway to affect GC cell migration, proliferation, metastasis and invasion (83). CDK2, a negative regulator of T cells, was determined to form a cross-network with lncRNAs and miRNAs integrating lncRNA-TF-mRNA and ceRNA networks related to *H. pylori*, regulating immune microenvironment with pathogenic roles of *H. pylori* (84). A ceRNA network constituted by the lncRNA-RP11-1094M14.8/miR-1269a/CXCL9 axis was revealed to be linked to a variety of immune cells *via* CXCL9, making it a potential target for GC with different degrees of immune cell infiltration (85).

LncRNAs can not only influence immune function directly by themselves but also affect the carcinogenesis of *H. pylori* by forming ceRNA networks with miRNAs to jointly regulate *H. pylori*-related tumor microenvironment.

## CircRNAs and *Helicobacter pylori*-related gastric cancer

CircRNAs are recognized to form a covalently closed loop structure by unique reverse splicing with the lack of terminal 5′ cap and 3′ polyadenylated tail (86). Due to their structural property, circRNAs have a high level of stability and tissue-specificity in physiological environment of eukaryotes. Most circRNAs are aberrantly expressed in pathological conditions such as cancer (87), which are also potential markers for disease progression.

The regulatory relationship of circRNAs with GC has been extensively studied. In recent years, circRNAs have shown promise as biomarkers for cancer diagnosis and prognosis especially for early cancer detection (88, 89). CircSOBP is closely associated with GC metastasis and poor survival of 5-year follow-up (90). CircARID1A regulates GC proliferation by forming an RNA-protein ternary complex with IGF2BP3 and SLC7A5, thus the circARID1A-IGF2BP3-SLC7A5 axis could be a novel therapeutic target for GC (91). The circ0008287/miR-548c-3p/CLIC1 axis promotes cell apoptosis and immune escape by impairing the function of CD8 + T cells in GC (92). CircEIF4G3 can inhibit GC cell proliferation and metastasis by regulating the miR-4449/SIK1 axis (93). Moreover, circ0002360 up-regulates PDLIM4 expression by sponging miR-629-3p (94). All these reports suggested the great potential of circRNAs to be prognostic biomarkers and therapeutic targets for GC.

However, the association of circRNAs with *H. pylori* and *H. pylori*-related GC remains rarely explored. Only a few studies presented that circRNAs might regulate the biological function or prognosis of *H. pylori*-related GC independently or by networks with miRNAs (95). *H. pylori* infection was found to increase circFNDC3B expression. In early gastric cancer (EGC) patients treated with endoscopic submucosal dissection (ESD), the expression of miR-942 and miR-510 was suppressed while their target genes CD44 and CDH1 were increased in the group with high circFNDC3B expression when compared with the low expression group, which contributed to a higher recurrence rate in EGC patients consequently (96). CD44 was considered as a stem cell-like cancer cell marker affecting EGC initiation with *H. pylori* infection (97). Earlier studies also confirmed that circFNDC3B could mediate GC cell migration and invasion by promoting epithelial-mesenchymal transition (EMT) (98). Additionally, *H. pylori* can upregulate circMAN1A2 expression in GC cells, and circMAN1A2 may promote proliferation, migration and invasion of *H. pylori*-induced GC by sponging miR-1236-3p to increase MTA2 expression (99).

In spite of the previous research on circRNAs associated with *H. pylori*-related GC, further investigations are needed to support circRNAs in serving as diagnostic biomarkers and therapeutic targets for *H. pylori*-related GC.

## Other ncRNAs and *Helicobacter pylori*-related gastric cancer

**FIGURE 2:**
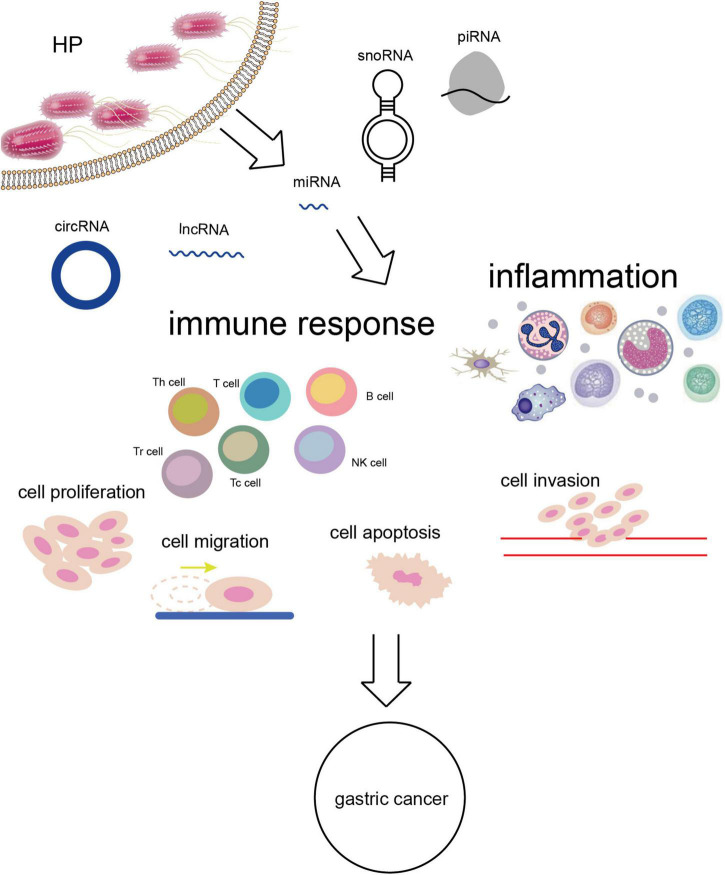
*H. pylori* infection alters the expression of ncRNAs leading to a series of biological effects *in vivo* and GC eventually.

Except for the highly studied miRNAs, lncRNAs, and circRNAs, ncRNAs also contain snoRNAs and piRNAs. The research on them could also be conducive to deeply understanding the association of ncRNAs with *H. pylori*-related GC although the known mechanisms are limited. *H. pylori* was suggested to be delivered into host cells by two snoRNAs enriched in outer membrane vesicles of bacteria including sR-2509025 and sR-989262, reduce LPS stimulation and inhibit IL-8 secretion, thereby mediating immune escape (100). The association of other ncRNAs with *H. pylori*-related GC worth further exploration (Figure 2).

## Summary

NcRNAs play critical roles in transcriptome with regulatory function in all aspects of physiological process, pathological process and disease progression. As one of the important pathogenic factors of GC, the association of *H. pylori* with ncRNAs has been extensively studied. NcRNAs have been clarified as a key link from persistent infection of *H. pylori* to GC and to make profound impacts despite the specific mechanisms to be confirmed. The regulation of ncRNAs on the immune microenvironment of body after *H. pylori* infection could be a therapeutic target for *H. pylori* in the future. The present review elaborated the bridge role of ncRNAs between *H. pylori* and GC from the perspective of immune inflammatory response, indicating that ncRNAs held promise as biomarkers for the early diagnosis, prognosis, and treatment of *H. pylori*-related GC. However, relevant exact molecular mechanisms need to be verified and more clinical data should be involved as additional evidence to the prognosis study, aiming to improve the survival and life quality of *H. pylori*-related GC patients.

## Author contributions

B-GW, Y-KL, and ZL conceived and designed this study and revised the manuscript. A-RL and L-YJ collected the data for the review. A-RL and Z-WY wrote the manuscript. All authors contributed to the article and approved the submitted version.

## References

[1] Sung H Ferlay J Siegel RL Laversanne M Soerjomataram I Jemal A Global cancer statistics 2020: GLOBOCAN estimates of incidence and mortality worldwide for 36 Cancers in 185 countries. *CA Cancer J Clin.* (2021) 71:209–49. 33538338 10.3322/caac.21660

[2] Wang F Meng W Wang B Qiao L. *Helicobacter* Pylori-induced gastric inflammation and gastric cancer. *Cancer Lett.* (2014) 345:196–202. 23981572 10.1016/j.canlet.2013.08.016

[3] Crowe SE. *Helicobacter* pylori infection. *N Engl J Med.* (2019) 380:1158–65. 30893536 10.1056/NEJMcp1710945

[4] Bravo D Hoare A Soto C Valenzuela MA Quest AF. *Helicobacter* pylori in human health and disease: mechanisms for local gastric and systemic effects. *World J Gastroenterol.* (2018) 24:3071–89. 10.3748/wjg.v24.i28.3071 30065554 PMC6064966

[5] Diaz P Valenzuela Valderrama M Bravo J Quest AFG. *Helicobacter* pylori and gastric cancer: adaptive cellular mechanisms involved in disease progression. *Front Microbiol.* (2018) 9:5. 10.3389/fmicb.2018.00005 29403459 PMC5786524

[6] Bagheri N Azadegan-Dehkordi F Rafieian-Kopaei M Rahimian G Asadi-Samani M Shirzad H. Clinical relevance of *Helicobacter* pylori virulence factors in Iranian patients with gastrointestinal diseases. *Microb Pathog.* (2016) 100:154–62. 27666510 10.1016/j.micpath.2016.09.016

[7] Blosse A Levy M Robe C Staedel C Copie-Bergman C Lehours P. Deregulation of miRNA in *Helicobacter* pylori-induced gastric MALT lymphoma: from mice to human. *J Clin Med.* (2019) 8:845. 10.3390/jcm8060845 31200531 PMC6616415

[8] Espinoza JL Matsumoto A Tanaka H Matsumura I. Gastric microbiota: an emerging player in *Helicobacter* pylori-induced gastric malignancies. *Cancer Lett.* (2018) 414:147–52. 10.1016/j.canlet.2017.11.009 29138097

[9] Suzuki M Mimuro H Kiga K Fukumatsu M Ishijima N Morikawa H *Helicobacter* pylori CagA phosphorylation-independent function in epithelial proliferation and inflammation. *Cell Host Microbe.* (2009) 5:23–34. 10.1016/j.chom.2008.11.010 19154985

[10] Deng J Miller SA Wang HY Xia W Wen Y Zhou BP beta-catenin interacts with and inhibits NF-kappa B in human colon and breast cancer. *Cancer Cell.* (2002) 2:323–34. 12398896 10.1016/s1535-6108(02)00154-x

[11] Supajatura V Ushio H Wada A Yahiro K Okumura K Ogawa H Cutting edge: VacA, a vacuolating cytotoxin of *Helicobacter* pylori, directly activates mast cells for migration and production of proinflammatory cytokines. *J Immunol.* (2002) 168:2603–7. 10.4049/jimmunol.168.6.2603 11884423

[12] Amedei A Cappon A Codolo G Cabrelle A Polenghi A Benagiano M The neutrophil-activating protein of *Helicobacter* pylori promotes Th1 immune responses. *J Clin Invest.* (2006) 116:1092–101. 10.1172/JCI27177 16543949 PMC1401483

[13] Toh JWT Wilson RB. Pathways of gastric carcinogenesis, *Helicobacter* pylori virulence and interactions with antioxidant systems, vitamin C and phytochemicals. *Int J Mol Sci.* (2020) 21:6451. 10.3390/ijms21176451 32899442 PMC7503565

[14] Fehlings M Drobbe L Moos V Renner Viveros P Hagen J Beigier-Bompadre M Comparative analysis of the interaction of *Helicobacter* pylori with human dendritic cells, macrophages, and monocytes. *Infect Immun.* (2012) 80:2724–34. 10.1128/IAI.00381-12 22615251 PMC3434561

[15] Adams BD Parsons C Walker L Zhang WC Slack FJ. Targeting noncoding RNAs in disease. *J Clin Invest.* (2017) 127:761–71. 28248199 10.1172/JCI84424 PMC5330746

[16] Deveson IW Hardwick SA Mercer TR Mattick JS. The dimensions, dynamics, and relevance of the mammalian noncoding transcriptome. *Trends Genet.* (2017) 33:464–78. 10.1016/j.tig.2017.04.004 28535931

[17] Friedman RC Farh KK Burge CB Bartel DP. Most mammalian mRNAs are conserved targets of microRNAs. *Genome Res.* (2009) 19:92–105. 18955434 10.1101/gr.082701.108 PMC2612969

[18] Rupaimoole R Slack FJ. MicroRNA therapeutics: towards a new era for the management of cancer and other diseases. *Nat Rev Drug Discov.* (2017) 16:203–22. 10.1038/nrd.2016.246 28209991

[19] Qi X Lin Y Chen J Shen B. Decoding competing endogenous RNA networks for cancer biomarker discovery. *Brief Bioinform.* (2020) 21:441–57. 10.1093/bib/bbz006 30715152

[20] Ye J Li J Zhao P. Roles of ncRNAs as ceRNAs in gastric cancer. *Genes (Basel).* (2021) 12:1036. 10.3390/genes12071036 PMC8305186 34356052

[21] Esquela-Kerscher A Slack FJ. Oncomirs - microRNAs with a role in cancer. *Nat Rev Cancer.* (2006) 6:259–69. 16557279 10.1038/nrc1840

[22] Banerjee S Thompson WE Chowdhury I. Emerging roles of microRNAs in the regulation of toll-like receptor (TLR)-signaling. *Front Biosci (Landmark Ed).* (2021) 26:771–96. 10.2741/4917 33049693 PMC9550351

[23] Huang WT Kuo SH Kuo YC Lin CW. miR-155-regulated mTOR and toll-like receptor 5 in gastric diffuse large B-cell lymphoma. *Cancer Med.* (2022) 11:555–70. 10.1002/cam4.4466 34913612 PMC8817081

[24] Pernitzsch SR Alzheimer M Bremer BU Robbe-Saule M De Reuse H Sharma CM. Small RNA mediated gradual control of lipopolysaccharide biosynthesis affects antibiotic resistance in *Helicobacter* pylori. *Nat Commun.* (2021) 12:4433. 10.1038/s41467-021-24689-2 34290242 PMC8295292

[25] Zhu XL Ren LF Wang HP Bai ZT Zhang L Meng WB Plasma microRNAs as potential new biomarkers for early detection of early gastric cancer. *World J Gastroenterol.* (2019) 25:1580–91. 30983818 10.3748/wjg.v25.i13.1580 PMC6452233

[26] Ghafouri-Fard S Vafaee R Shoorei H Taheri M. MicroRNAs in gastric cancer: biomarkers and therapeutic targets. *Gene.* (2020) 757:144937. 10.1016/j.gene.2020.144937 32640300

[27] Vinchure OS Kulshreshtha R. miR-490: a potential biomarker and therapeutic target in cancer and other diseases. *J Cell Physiol.* (2021) 236:3178–93. 10.1002/jcp.30119 33094503

[28] ZiaSarabi P Sorayayi S Hesari A Ghasemi F. Circulating microRNA-133, microRNA-17 and microRNA-25 in serum and its potential diagnostic value in gastric cancer. *J Cell Biochem.* (2019) 120:12376–81. 10.1002/jcb.28503 30861177

[29] Link A Schirrmeister W Langner C Varbanova M Bornschein J Wex T Differential expression of microRNAs in preneoplastic gastric mucosa. *Sci Rep.* (2015) 5:8270. 10.1038/srep08270 PMC4317705 25652892

[30] Ou Y Ren H Zhao R Song L Liu Z Xu W *Helicobacter* pylori CagA promotes the malignant transformation of gastric mucosal epithelial cells through the dysregulation of the miR-155/KLF4 signaling pathway. *Mol Carcinog.* (2019) 58:1427–37. 10.1002/mc.23025 31162747

[31] Wan J Xia L Xu W Lu N. Expression and function of miR-155 in diseases of the gastrointestinal tract. *Int J Mol Sci.* (2016) 17:709. 10.3390/ijms17050709 PMC4881531 27187359

[32] Pachathundikandi SK Blaser N Backert S. Mechanisms of inflammasome signaling, microRNA induction and resolution of inflammation by *Helicobacter* pylori. *Curr Top Microbiol Immunol.* (2019) 421:267–302. 10.1007/978-3-030-15138-6_11 31123893

[33] Mahbobi R Fallah F Behmanesh A Yadegar A Hakemi-Vala M Ehsanzadeh SJ *Helicobacter* pylori infection mediates inflammation and tumorigenesis-associated genes through miR-155-5p: an integrative omics and bioinformatics-based investigation. *Curr Microbiol.* (2022) 79:192. 10.1007/s00284-022-02880-y 35551487

[34] Fassi Fehri L Koch M Belogolova E Khalil H Bolz C Kalali B *Helicobacter* pylori induces miR-155 in T cells in a cAMP-Foxp3-dependent manner. *PLoS One.* (2010) 5:e9500. 10.1371/journal.pone.0009500 20209161 PMC2830477

[35] Wang J Deng Z Wang Z Wu J Gu T Jiang Y MicroRNA-155 in exosomes secreted from *Helicobacter* pylori infection macrophages immunomodulates inflammatory response. *Am J Transl Res.* (2016) 8:3700–9. 27725852 PMC5040670

[36] Wu K Zhu C Yao Y Wang X Song J Zhai J. MicroRNA-155-enhanced autophagy in human gastric epithelial cell in response to *Helicobacter* pylori. *Saudi J Gastroenterol.* (2016) 22:30–6. 10.4103/1319-3767.173756 26831604 PMC4763526

[37] Oertli M Engler DB Kohler E Koch M Meyer TF Muller A. MicroRNA-155 is essential for the T cell-mediated control of *Helicobacter* pylori infection and for the induction of chronic gastritis and colitis. *J Immunol.* (2011) 187:3578–86. 10.4049/jimmunol.1101772 21880981

[38] Koch M Mollenkopf HJ Klemm U Meyer TF. Induction of microRNA-155 is TLR- and type IV secretion system-dependent in macrophages and inhibits DNA-damage induced apoptosis. *Proc Natl Acad Sci USA.* (2012) 109:E1153–62. 10.1073/pnas.1116125109 22509021 PMC3358876

[39] Xiao B Liu Z Li BS Tang B Li W Guo G Induction of microRNA-155 during *Helicobacter* pylori infection and its negative regulatory role in the inflammatory response. *J Infect Dis.* (2009) 200:916–25. 10.1086/605443 19650740

[40] Tang B Xiao B Liu Z Li N Zhu ED Li BS Identification of MyD88 as a novel target of miR-155, involved in negative regulation of *Helicobacter* pylori-induced inflammation. *FEBS Lett.* (2010) 584:1481–6. 10.1016/j.febslet.2010.02.063 20219467

[41] Liu TY Chen SU Kuo SH Cheng AL Lin CW. E2A-positive gastric MALT lymphoma has weaker plasmacytoid infiltrates and stronger expression of the memory B-cell-associated miR-223: possible correlation with stage and treatment response. *Mod Pathol.* (2010) 23:1507–17. 10.1038/modpathol.2010.139 20802470

[42] Saha K Sarkar D Khan U Karmakar BC Paul S Mukhopadhyay AK Capsaicin inhibits inflammation and gastric damage during H pylori infection by targeting NF-kB-miRNA Axis. *Pathogens.* (2022) 11:641. 10.3390/pathogens11060641 35745495 PMC9227394

[43] Vasapolli R Venerito M Schirrmeister W Thon C Weigt J Wex T Inflammatory microRNAs in gastric mucosa are modulated by *Helicobacter* pylori infection and proton-pump inhibitors but not by aspirin or NSAIDs. *PLoS One.* (2021) 16:e0249282. 10.1371/journal.pone.0249282 33857171 PMC8049315

[44] Yang F Xu Y Liu C Ma C Zou S Xu X NF-kappaB/miR-223-3p/ARID1A axis is involved in *Helicobacter* pylori CagA-induced gastric carcinogenesis and progression. *Cell Death Dis.* (2018) 9:12. 10.1038/s41419-017-0020-9 29317648 PMC5849037

[45] Pachathundikandi SK Backert S. *Helicobacter* pylori controls NLRP3 expression by regulating hsa-miR-223-3p and IL-10 in cultured and primary human immune cells. *Innate Immun.* (2018) 24:11–23. 10.1177/1753425917738043 29145789 PMC6830764

[46] Matsushima K Isomoto H Inoue N Nakayama T Hayashi T Nakayama M MicroRNA signatures in *Helicobacter* pylori-infected gastric mucosa. *Int J Cancer.* (2011) 128:361–70. 20333682 10.1002/ijc.25348

[47] Zingone F Pilotto V Cardin R Maddalo G Orlando C Fassan M Autoimmune atrophic gastritis: the role of miRNA in relation to *Helicobacter* pylori infection. *Front Immunol.* (2022) 13:930989. 10.3389/fimmu.2022.930989 35941891 PMC9356369

[48] Gong R Han R Zhuang X Tang W Xu G Zhang L MiR-375 mitigates retinal angiogenesis by depressing the JAK2/STAT3 pathway. *Aging (Albany NY).* (2022) 14:6594–604. 10.18632/aging.204232 35980290 PMC9467412

[49] Li L Xiao C He K Xiang G. Circ_0072088 promotes progression of hepatocellular carcinoma by activating JAK2/STAT3 signaling pathway via miR-375. *IUBMB Life.* (2021) 73:1153–65. 10.1002/iub.2520 34148288

[50] Yan XL Luo QY Zhou SN Pan WT Zhang L Yang DJ MicroRNA-375 reverses the expression of PD-L1 by inactivating the JAK2/STAT3 signaling pathways in gastric cancer. *Clin Res Hepatol Gastroenterol.* (2021) 45:101574. 10.1016/j.clinre.2020.10.015 33272890

[51] Zhao Q Liu Y Wang T Yang Y Ni H Liu H MiR-375 inhibits the stemness of breast cancer cells by blocking the JAK2/STAT3 signaling. *Eur J Pharmacol.* (2020) 884:173359. 10.1016/j.ejphar.2020.173359 32738343

[52] Zhang Z Chen S Fan M Ruan G Xi T Zheng L *Helicobacter* pylori induces gastric cancer via down-regulating miR-375 to inhibit dendritic cell maturation. *Helicobacter*. (2021) 26:e12813. 10.1111/hel.12813 33938607

[53] Miao L Liu K Xie M Xing Y Xi T. miR-375 inhibits *Helicobacter* pylori-induced gastric carcinogenesis by blocking JAK2-STAT3 signaling. *Cancer Immunol Immunother.* (2014) 63:699–711. 10.1007/s00262-014-1550-y 24718681 PMC11028505

[54] Rossi AF Cadamuro AC Biselli-Perico JM Leite KR Severino FE Reis PP Interaction between inflammatory mediators and miRNAs in *Helicobacter* pylori infection. *Cell Microbiol.* (2016) 18:1444–58. 26945693 10.1111/cmi.12587 PMC5074252

[55] Li N Wang J Yu W Dong K You F Si B MicroRNA146a inhibits the inflammatory responses induced by interleukin17A during the infection of *Helicobacter* pylori. *Mol Med Rep.* (2019) 19:1388–95. 30535468 10.3892/mmr.2018.9725

[56] Oana SM Claudia B Lelia RA Simona M Claudia C Daniela DE. Differential expression of tissular miRNA-155 in pediatric gastritis. *J Clin Med.* (2022) 11:3351. 10.3390/jcm11123351 35743416 PMC9224896

[57] Cortes-Marquez AC Mendoza-Elizalde S Arenas-Huertero F Trillo-Tinoco J Valencia-Mayoral P Consuelo-Sanchez A Differential expression of miRNA-146a and miRNA-155 in gastritis induced by *Helicobacter* pylori infection in paediatric patients, adults, and an animal model. *BMC Infect Dis.* (2018) 18:463. 10.1186/s12879-018-3368-2 30219037 PMC6139157

[58] Chen P Guo H Wu X Li J Duan X Ba Q Epigenetic silencing of microRNA-204 by *Helicobacter* pylori augments the NF-kappaB signaling pathway in gastric cancer development and progression. *Carcinogenesis.* (2020) 41:430–41. 10.1093/carcin/bgz143 31873718

[59] Tsai CC Chen TY Tsai KJ Lin MW Hsu CY Wu DC NF-kappaB/miR-18a-3p and miR-4286/BZRAP1 axis may mediate carcinogenesis in *Helicobacter* pylori-associated gastric cancer. *Biomed Pharmacother.* (2020) 132:110869. 10.1016/j.biopha.2020.110869 33113427

[60] Gao C Zhang Z Liu W Xiao S Gu W Lu H. Reduced microRNA-218 expression is associated with high nuclear factor kappa B activation in gastric cancer. *Cancer.* (2010) 116:41–9. 10.1002/cncr.24743 19890957

[61] Zou M Wang F Jiang A Xia A Kong S Gong C MicroRNA-3178 ameliorates inflammation and gastric carcinogenesis promoted by *Helicobacter* pylori new toxin, Tip-alpha, by targeting TRAF3. *Helicobacter.* (2017) 22:e12348. 10.1111/hel.12348 27493095

[62] Teng GG Wang WH Dai Y Wang SJ Chu YX Li J. Let-7b is involved in the inflammation and immune responses associated with *Helicobacter* pylori infection by targeting Toll-like receptor 4. *PLoS One.* (2013) 8:e56709. 10.1371/journal.pone.0056709 23437218 PMC3577724

[63] Ding L Li Q Chakrabarti J Munoz A Faure-Kumar E Ocadiz-Ruiz R MiR130b from Schlafen4(+) MDSCs stimulates epithelial proliferation and correlates with preneoplastic changes prior to gastric cancer. *Gut.* (2020) 69:1750–61. 10.1136/gutjnl-2019-318817 31980446 PMC7377952

[64] Zhao M Liu Q Liu W Zhou H Zang X Lu J. MicroRNA140 suppresses *Helicobacter* pyloripositive gastric cancer growth by enhancing the antitumor immune response. *Mol Med Rep.* (2019) 20:2484–92. 31322226 10.3892/mmr.2019.10475

[65] Xie G Li W Li R Wu K Zhao E Zhang Y *Helicobacter* pylori promote B7-H1 expression by suppressing miR-152 and miR-200b in gastric cancer cells. *PLoS One.* (2017) 12:e0168822. 10.1371/journal.pone.0168822 28056089 PMC5215825

[66] Kalani M Hodjati H Ghoddusi Johari H Doroudchi M. Memory T cells of patients with abdominal aortic aneurysm differentially expressed micro RNAs 21, 92a, 146a, 155, 326 and 663 in response to *Helicobacter* pylori and *Lactobacillus acidophilus*. *Mol Immunol.* (2021) 130:77–84. 10.1016/j.molimm.2020.11.007 33246580

[67] Zhu Y Liu L Hu L Dong W Zhang M Liu Y Effect of *Celastrus orbiculatus* in inhibiting *Helicobacter* pylori induced inflammatory response by regulating epithelial mesenchymal transition and targeting miR-21/PDCD4 signaling pathway in gastric epithelial cells. *BMC Complement Altern Med.* (2019) 19:91. 10.1186/s12906-019-2504-x 31035975 PMC6489279

[68] Murray-Stewart T Sierra JC Piazuelo MB Mera RM Chaturvedi R Bravo LE Epigenetic silencing of miR-124 prevents spermine oxidase regulation: implications for *Helicobacter* pylori-induced gastric cancer. *Oncogene.* (2016) 35:5480–8. 10.1038/onc.2016.91 27041578 PMC5050049

[69] Zheng L Wu Y Shen L Liang X Yang Z Li S Mechanisms of JARID1B Up-regulation and its role in *Helicobacter* pylori-induced gastric carcinogenesis. *Front Oncol.* (2021) 11:757497. 10.3389/fonc.2021.757497 34778074 PMC8581301

[70] Pagliari M Munari F Toffoletto M Lonardi S Chemello F Codolo G *Helicobacter* pylori affects the antigen presentation activity of macrophages modulating the expression of the immune receptor CD300E through miR-4270. *Front Immunol.* (2017) 8:1288. 10.3389/fimmu.2017.01288 29085364 PMC5649134

[71] Ishimoto T Izumi D Watanabe M Yoshida N Hidaka K Miyake K Chronic inflammation with *Helicobacter* pylori infection is implicated in CD44 overexpression through miR-328 suppression in the gastric mucosa. *J Gastroenterol.* (2015) 50:751–7. 10.1007/s00535-014-1019-y 25479940

[72] Li S Liang X Ma L Shen L Li T Zheng L MiR-22 sustains NLRP3 expression and attenuates H. pylori-induced gastric carcinogenesis. *Oncogene.* (2018) 37:884–96. 10.1038/onc.2017.381 29059152

[73] Sirey TM Roberts K Haerty W Bedoya-Reina O Rogatti-Granados S Tan JY The long non-coding RNA Cerox1 is a post transcriptional regulator of mitochondrial complex I catalytic activity. *Elife.* (2019) 8:e45051. 10.7554/eLife.45051 PMC6542586 31045494

[74] Delas MJ Sabin LR Dolzhenko E Knott SR Munera Maravilla E Jackson BT lncRNA requirements for mouse acute myeloid leukemia and normal differentiation. *Elife.* (2017) 6:e25607. 10.7554/eLife.25607 28875933 PMC5619947

[75] Xin Z Zhang L Liu M Wang Y Zhang Y Zhao W *Helicobacter* pylori infection-related long non-coding RNA signatures predict the prognostic status for gastric cancer patients. *Front Oncol.* (2021) 11:709796. 10.3389/fonc.2021.709796 34386426 PMC8353258

[76] Wang Y Huang L Wang Y Luo W Li F Xiao J Single-cell RNA-sequencing analysis identifies host long noncoding RNA MAMDC2-AS1 as a co-factor for HSV-1 nuclear transport. *Int J Biol Sci.* (2020) 16:1586–603. 10.7150/ijbs.42556 32226304 PMC7097924

[77] Jing XH Li LX Han TT Shi J. [Effects of long non-coding RNA plasmacytoma variant translocation 1 gene on inflammatory response and cell migration in *Helicobacter* pylori infected gastric epithelial cell line]. *Zhongguo Yi Xue Ke Xue Yuan Xue Bao.* (2020) 42:228–35. 32385030 10.3881/j.issn.1000-503X.11395

[78] Zhang Y Yan J Li C Wang X Dong Y Shen X LncRNA H19 induced by *Helicobacter* pylori infection promotes gastric cancer cell growth via enhancing NF-kappaB-induced inflammation. *J Inflamm (Lond).* (2019) 16:23. 10.1186/s12950-019-0226-y 31787851 PMC6878690

[79] Mohamed WA Schaalan MF Ramadan B. The expression profiling of circulating miR-204, miR-182, and lncRNA H19 as novel potential biomarkers for the progression of peptic ulcer to gastric cancer. *J Cell Biochem.* (2019) 120:13464–77. 10.1002/jcb.28620 30945348

[80] Yao Y Jiang Q Jiang L Wu J Zhang Q Wang J Lnc-SGK1 induced by *Helicobacter* pylori infection and highsalt diet promote Th2 and Th17 differentiation in human gastric cancer by SGK1/Jun B signaling. *Oncotarget.* (2016) 7:20549–60. 10.18632/oncotarget.7823 26942879 PMC4991474

[81] Zhang J Wei J Wang Z Feng Y Wei Z Hou X Transcriptome hallmarks in *Helicobacter* pylori infection influence gastric cancer and MALT lymphoma. *Epigenomics.* (2020) 12:661–71. 10.2217/epi-2019-0152 32129675

[82] Shafiee M Aleyasin SA Mowla SJ Vasei M Yazdanparast SA. The effect of MicroRNA-375 overexpression, an inhibitor of *Helicobacter* pylori-induced carcinogenesis, on lncRNA SOX2OT. *Jundishapur J Microbiol.* (2016) 9:e23464. 10.5812/jjm.23464 27800139 PMC5081003

[83] Qu F Zhu B Hu YL Mao QS Feng Y. LncRNA HOXA-AS3 promotes gastric cancer progression by regulating miR-29a-3p/LTbetaR and activating NF-kappaB signaling. *Cancer Cell Int.* (2021) 21:118. 10.1186/s12935-021-01827-w 33602223 PMC7890634

[84] Liu S Yin H Zheng S Chu A Li Y Xing C Differentially expressed mRNAs and their long noncoding RNA regulatory network with *Helicobacter* pylori-associated diseases including atrophic gastritis and gastric cancer. *Biomed Res Int.* (2020) 2020:3012193. 10.1155/2020/3012193 33282942 PMC7686847

[85] Zhang K Zhang L Mi Y Tang Y Ren F Liu B A ceRNA network and a potential regulatory axis in gastric cancer with different degrees of immune cell infiltration. *Cancer Sci.* (2020) 111:4041–50. 10.1111/cas.14634 32860283 PMC7648034

[86] Chen LL Yang L. Regulation of circRNA biogenesis. *RNA Biol.* (2015) 12:381–8. 25746834 10.1080/15476286.2015.1020271 PMC4615371

[87] Bach DH Lee SK Sood AK. Circular RNAs in cancer. *Mol Ther Nucleic Acids.* (2019) 16:118–29. 30861414 10.1016/j.omtn.2019.02.005 PMC6411617

[88] Kristensen LS Jakobsen T Hager H Kjems J. The emerging roles of circRNAs in cancer and oncology. *Nat Rev Clin Oncol.* (2022) 19:188–206. 34912049 10.1038/s41571-021-00585-y

[89] Ghafouri-Fard S Honarmand Tamizkar K Jamali E Taheri M Ayatollahi SA. Contribution of circRNAs in gastric cancer. *Pathol Res Pract.* (2021) 227:153640. 10.1016/j.prp.2021.153640 34624593

[90] Yan X Wang J Bai Y. Potentials of circSOBP in the diagnosis and prognosis of gastric cancer. *Scand J Gastroenterol.* (2022):1–5. 10.1080/00365521.2022.2088246 35993263

[91] Ma Q Yang F Huang B Pan X Li W Yu T CircARID1A binds to IGF2BP3 in gastric cancer and promotes cancer proliferation by forming a circARID1A-IGF2BP3-SLC7A5 RNA-protein ternary complex. *J Exp Clin Cancer Res.* (2022) 41:251. 10.1186/s13046-022-02466-3 35986300 PMC9389715

[92] Li B Liang L Chen Y Liu J Wang Z Mao Y Circ_0008287 promotes immune escape of gastric cancer cells through impairing microRNA-548c-3p-dependent inhibition of CLIC1. *Int Immunopharmacol.* (2022) 111:108918. 10.1016/j.intimp.2022.108918 35905561

[93] Zang X Jiang J Gu J Chen Y Wang M Zhang Y Circular RNA EIF4G3 suppresses gastric cancer progression through inhibition of beta-catenin by promoting delta-catenin ubiquitin degradation and upregulating SIK1. *Mol Cancer.* (2022) 21:141. 10.1186/s12943-022-01606-9 35780119 PMC9250212

[94] Yu Z Lan J Li W Jin L Qi F Yu C Circular RNA hsa_circ_0002360 promotes proliferation and invasion and inhibits oxidative stress in gastric cancer by sponging miR-629-3p and regulating the PDLIM4 expression. *Oxid Med Cell Longev.* (2022) 2022:2775433. 10.1155/2022/2775433 35982735 PMC9381216

[95] Wu Z Liu P Zhang G. Identification of circRNA-miRNA-immune-related mRNA regulatory network in gastric cancer. *Front Oncol.* (2022) 12:816884. 10.3389/fonc.2022.816884 35280778 PMC8907717

[96] Zhang J Bai J Zhu H Li W An Q Wang D. The upregulation of circFNDC3B aggravates the recurrence after endoscopic submucosal dissection (ESD) in early gastric cancer (EGC) patients. *Sci Rep.* (2022) 12:6178. 10.1038/s41598-022-07154-y 35418175 PMC9007947

[97] Takaishi S Okumura T Tu S Wang SS Shibata W Vigneshwaran R Identification of gastric cancer stem cells using the cell surface marker CD44. *Stem Cells.* (2009) 27:1006–20. 19415765 10.1002/stem.30 PMC2746367

[98] Hong Y Qin H Li Y Zhang Y Zhuang X Liu L FNDC3B circular RNA promotes the migration and invasion of gastric cancer cells via the regulation of E-cadherin and CD44 expression. *J Cell Physiol.* (2019) 234:19895–910. 10.1002/jcp.28588 30963578 PMC6766960

[99] Guo R Cui X Li X Zang W Chang M Sun Z CircMAN1A2 is upregulated by *Helicobacter* pylori and promotes development of gastric cancer. *Cell Death Dis.* (2022) 13:409. 10.1038/s41419-022-04811-y 35484118 PMC9051101

[100] Zhang H Zhang Y Song Z Li R Ruan H Liu Q sncRNAs packaged by *Helicobacter* pylori outer membrane vesicles attenuate IL-8 secretion in human cells. *Int J Med Microbiol.* (2020) 310:151356. 10.1016/j.ijmm.2019.151356 31585715

